# Congenital transmission of Chagas disease in a non-endemic area, is an early diagnosis possible?

**DOI:** 10.1371/journal.pone.0218491

**Published:** 2019-07-10

**Authors:** Laura Francisco-González, Alba Rubio-San-Simón, María Isabel González-Tomé, Ángela Manzanares, Cristina Epalza, María del Mar Santos, Teresa Gastañaga, Paloma Merino, José Tomás Ramos-Amador

**Affiliations:** 1 Department of Pediatrics, Hospital Clínico San Carlos, Complutense University of Madrid, Madrid, Spain; 2 Department of Pediatrics, Hospital Doce de Octubre, Madrid, Spain; 3 Department of Pediatrics, Hospital Gregorio Marañón, Madrid, Spain; 4 Department of Obstetrics and Gynaecology, Hospital Clínico San Carlos, Madrid, Spain; 5 Department of Microbiology, Hospital Clínico San Carlos, Madrid, Spain; 6 Department of Pediatrics, Hospital Clínico San Carlos, Instituto de Investigación Sanitaria del Hospital Clínico San Carlos (IdISSC), Complutense University of Madrid, Madrid, Spain; National Institute for Infectious Diseases IRCCS Lazzaro Spallanzani, ITALY

## Abstract

**Background:**

Chagas disease (CD) is an emergent disease in Europe, due to immigration. The aims of this study are to describe the epidemiological characteristics of a cohort of Chagas infected pregnant women in Spain, to assess the vertical transmission (VT) rate and evaluate the usefulness of the PCR in the diagnosis of congenital infection in the first months of life.

**Methods:**

A descriptive, retrospective study including Chagas seropositive pregnant women who were attended at three tertiary hospitals in Madrid, from January 2012 to September 2016. Infants were examined by PCR at birth and 1 month later and serologically studied at 9 months or later. Children were considered infected when the parasite was detected by PCR at any age or when serology remained positive without decline over the age of 9 months.

**Results:**

We included 122 seropositive-infected pregnant women, 81% were from Bolivia and only 8.2% had been treated before. 125 newborns were studied and finally 109 were included (12.8% lost the follow-up before performing the last serology). The VT rate was 2.75% (95% CI: 0,57–8,8%). Infected infants had positive PCR at birth and 1 month later. All of them were treated successfully with benznidazole (PCR and serology became negative later on). All non-infected children presented negative PCR. The mean age at which uninfected patients had negative serology was 10.5 months.

**Conclusions:**

The VT rate is in keeping with literature and confirms the need to carry out a screening in pregnant women coming from endemic areas. PCR seems to be a useful tool to provide early diagnosis of congenital CD.

## Introduction

Over the past years there has been an increase in migration flows between different geographical areas, causing variations in epidemiological patterns of diseases worldwide. Chagas disease (CD), caused by the protozoan *Trypanosoma cruzi*, has traditionally been confined to endemic locations in South America, but nowadays is emerging in Europe[1], mainly in Spain, the European country with the highest immigration rate from South America and with the highest number of individuals with CD [2]. Most of new cases of CD in Spain occur through vertical mother-to-child transmission (VT).

Murcia L. et al [3] have recently described the effectiveness of treating infected women to prevent congenital CD. The risk of congenital transmission seems to be related to maternal *Trypanosoma* parasitemia, suggesting that early treatment should be performed in infected women of childbearing age in order to prevent congenital CD. In addition, an early diagnosis is essential as younger infants have better response to treatment [4].

In the last years, a significant effort has been made in Spain to establish a Chagas screening program in pregnant mothers native from endemic areas. For diagnosis of congenital CD in children born to infected mothers Spanish guidelines recommend performing: Polymerase Chain-Reaction (PCR) and /or microhaematocrit detection at birth and 1 month later, and a serological determination at 9 months of age or later[4]. Nowadays, this screening is not universal and therefore misdiagnosis of VT cases may occur. Furthermore, as children born to infected mothers need to be followed up for a prolonged period until the confirmation of a negative serology, a significant proportion of parents may be loss to follow up before the last serology is performed and eventually might be infected.

The aims of this study were to describe the epidemiological characteristics of a cohort of Chagas infected pregnant women in our country, to assess the VT rate and evaluate the usefulness of the PCR in the diagnosis of VT of *T*.*cruzi* in the first months of life. We purpose that this deep analysis could help to evaluate ways to improve the newborn screening program in our setting.

## Patients and methods

We conducted a descriptive, retrospective study including Chagas seropositive pregnant women who were attended at three tertiary hospitals in Madrid (Spain) and their newborns, from January 2012 to September 2016. Inclusion criteria: all pregnant women with a positive serology performed during pregnancy and follow-up completed for the first year of their newborns.

The study was reviewed and approved by the Ethics Committee of the Hospital Clínico San Carlos (January 2017), because it has competence over the three participant hospitals. An informed consent was not requested to collect the data for the study because it is a retrospective, observational, risk-free study for patients whose identity was anonymized. The data were treated confidentially in accordance with current local, national and international legislation.

A serologic assay (enzyme-linked immunosorbent assay or chemiluminescence analysis, depending on the hospital) was performed in pregnant women from endemic areas (between Mexico and Argentina, except the Caribbean Islands) for Trypanosoma cruzi screening. Positive cases were confirmed with immunochromatograpy. In cases of discrepancy indirect immunofluorescence was conducted.

Newborn follow-up was carried out following the recommendations of the Spanish Society of Pediatric Infectious Diseases (Sociedad Española de Infectología Pediátrica, SEIP), the Spanish Society of Clinical Microbiology (Sociedad Española de Microbiología Clínica, SEIMC) and the Spanish Society of Gynecology and Obstetrics (Sociedad Española de Ginecología yObstetricia, SEGO)[4]. A PCR at birth and 1 month later, and a serological determination at 9 months of age or later were performed. Children were considered infected when the parasite was detected by PCR at any age (confirmed in a second determination) or when serology remained positive without decrease over 9 months-old of age. The infection was ruled out when the serology became negative. A second sample at 12 months was required to all children with positive serology at 9 months to confirm or rule out the infection. The time of negativization of *T*.*cruzi* antibodies was considered at the moment when the first negative serology was obtained. The PCR study was performed with an in-house standardized PCR based on Seringer et al and Norman et al protocol [5][6] in two of the hospitals and with a Progenie comercial kit (RealCyclerCHAG) in the third one, with a qualitative (positive or negative) analysis.

Clinical and epidemiological data were collected. The stage of CD and the moment when previous treatment was given has not systematically been registered in the database in all mothers.

A data abstraction form was created *a priori* and information relevant to the study research question was extracted independently by three investigators. Where there were data discrepancies, the investigators met for discussion until a consensus was made. All the results were analyzed using the IBM SPSS Statistics software.

The results of the quantitative variables will be expressed by their mean and standard deviation. For qualitative variables, the results will be expressed by their frequencies and percentages. The congenital transmission rate was calculated as the number of congenitally infected infants divided by the number of infants born to infected mothers, excluding patients in whom the follow-up was lost.

## Results

We included 122 seropositive-infected pregnant women, who ranged in age from 19 to 42 (mean age ± SD, 32.6 ± 4.3 years). Ninety-nine (81.1%) were from Bolivia, 14 (11.5%) from Paraguay, 2 (1.6%) from Argentina, 2 (1.6%) from Honduras and the rest from different Latin American countries. Among the 122 women, 10 (8.2%) had been treated before current pregnancy.

A total of 125 infants (52% girls, 48% boys) from 122 pregnancies were initially included but 12.8% lost the follow-up before performing the last serology, thus only 109 infants completed the follow-up beyond 12 months, and are included in the analysis of the VT rate.

Vertical transmission was detected in 3 newborns, which represents a congenital transmission rate of 2.75% (95% CI: 0,57–8,8%). None of these 3 mothers received treatment before the pregnancy. Only one infant had a case of symptomatic congenital Chagas disease (hydrops fetalis, ascites, haemodynamic inestability, anaemia) and required admission to the neonatal intensive care unit [7]. All infected infants had PCR at birth and at 1-month-old positive. After treatment PCR became negative. In all three cases serological test was initially also positive and became negative after the age of 12 months. Infected infants were treated with benznidazole. Two of the patients were treated at one month of age, once we had two positive PCR; but in the symptomatic case the treatment was initiated when the positive result of the first PCR performed (at birth) was known, however after one month he had not completed the treatment and the PCR remained positive. Side effects were observed only in one patient who suffered moderate neutropenia (nadir: 600cel/mcl). This patient required a reduction of the dose of benznidazole from 6mg/Kg/day to 5mg/Kg/day to resolve it.

Findings in infants with congenital infection are summarized in Table 1.

**Table 1:** Characteristics of the 3 newborns with congenital Chagas infection.

	Newborn 1	Newborn 2	Newborn 3
Maternal country	Bolivia	Bolivia	Bolivia
Maternal age (years)	25	26	38
Maternal disease status	Chronic	Chronic	Chronic
Pregnancy	Singleton	Singleton	Singleton
Gestational age (weeks)	38+2	38+3	36+6
Mode of delivery	Vaginal—instrument assisted	Vaginal—spontaneous	Elective caesarian
Gender	Female	Male	Male
Clinical manifestations at birth	No	No	Yes
PCR at birth	Positive	Positive	Positive
PCR at 1 month of life	Positive	Positive	Positive
Treatment	Benznidazole	Benznidazole	Benznidazole
Treatment dose (mg/Kg/day)	5	10	10
Treatment age of onset (days)	45	30	15
Length of treatment (days)	49	60	60
Secondary effects	Moderate neutropenia	-	-
1st Serology (Age months /result)	9 / Positive	9 / Positive	3 / Positive
2nd Serology (Age months /result)	12.9 / Negative	15.6 / Negative	3.8 / Positive
3rd Serology (Age months /result)	-	-	20.2 / Negative
PCR post-treatment (Age months /result)	12.9 / Negative	9.9/ Negative	3 / Negative
Total blood samples obtained	4	5	6

In contrast, no transmission was observed in 106 newborns, all of them with a negative PCR result at birth and at 1 month of life. In all these infants, serological test became negative during follow-up.

It should be noted that PCR did not report any false positive nor false negative in our sample. Therefore, the concordance between the determination of PCR at birth and at 1 month later was 100%.

The first serology was done on average at 9.7 months of age (SD 1,45) but the mean age at which uninfected patients had negative serology was 10.5 months (SD 2). The range of age the serology was done was between 7,5–20,2 months.

The number of serology tests performed per patient varied from 1 to 4, and 20% of infants required at least 2 determinations before a negative result was obtained.

## Discussion

In our study most pregnant women with positive serology for *T*.*cruzi* (81%) came from Bolivia, the country in Latin America with the highest prevalence of CD. The rate of VT in our cohort was 2.75% (95% CI, 0,57–8,8%), according to the data reported in the literature [8][9], where the referred prevalence is estimated at approximately 5% of newborns of infected mothers in endemic areas and 2–3% in non-endemic areas.

The low observed transmission rate in our study compared to endemic areas, although with a wide confident interval, might be influenced by the chronic phase of the disease in pregnant women as they were probably infected in the childhood.

It’s important that in our study only the 8,2% of women had received treatment for CD, and in the three cases in which we have documented VT, mothers had not received treatment before. This disease in endemic areas affects mainly low socioeconomic population; in many cases it is not diagnosed or even when the diagnosis is known, the treatment is not administered or completed because it is usually poorly tolerated, specially in adults. The long asymptomatic acute phase contributes to this underdiagnosis [2][10]. The ideal strategy to avoid this situation would be to screen people from endemic areas in primary care, especially women of childbearing age as an effective measure to reduce VT.

The fact that pregnant women present positive PCR during pregnancy increases the risk of VT compared to women with positive serology but negative PCR. Murcia L. et al [3] propose to include PCR in the management of pregnant women with Chagas disease, as they find a NPV of 100%; with which its negativity indicates a low risk of VT, while its positivity would force a closer monitoring of the newborn.

Congenital CD is usually asymptomatic. For patients presenting with symptoms, these are variable and usually involve several organs and systems, as in the case of the symptomatic VT identified in our cohort [7][11][12].

A positive serologic test in newborns can result from the placental transfer of maternal antibodies, so the diagnosis should be based on methods for parasite detection (PCR or blood smear examination), which are recommended to perform at birth and repeating at age 1 month due to the possibility of false negative results (depending on the level of parasitaemia). In Spain, the use of PCR is more extended because is easier to perform, and it does not depend on the experience of the laboratory staff compared to blood smear (). In our study, microhaematocrit was only performed in 30,4% of the sample (38/125). In this group, all the patients have a negative result. In the three cases of congenital transmission only one has the both determinations (PCR and microhaematocrit), in which the microhaematocrit result was negative in spite of a positive PCR. In Spain, the use of PCR is recommended due to its higher sensitivity compared to blood smear (observation of the parasite by microscopic examination the smear). The sensitivity of PCR is high during the acute phase (up to 90–95%) and decreases during the chronic phase of disease (ranging between 50% and 80% in different case series)[13, 14]; furthermore, it has a specificity of nearly 100% [13]. Nevertheless, microscopic examination of blood smears continues to be useful in endemic areas, where molecular techniques such as PCR are less frequently available in laboratories.

When getting a positive PCR, it is recommended proceed with a second determination, to confirm the result. The case of a neonate with discordant results (a positive PCR unconfirmed in a new determination) would be controversial. We have not come across such a case in our series but, in this situation,a false positive result could be possible due to transplacental transfer and transient persistence of maternal parasite DNA [15]. A third determination could be made (and if possible also microhaematocrit) and if they were also negative, in an asymptomatics patient, it would be possible to wait without treatment and verify later a decrease of the antibody titers over time. If there is no decrease, initiation of treatment should be considered.

In our case, PCR might improve the diagnostic yield if a higher sensitivity is confirmed. The main drawback is the lack of standardization of the procedure and therefore it has not been definitely validated for diagnosis of congenital CD. Despite these shortcomings, in our study, PCR has an excellent sensitivity to detect early CD in all 3 infected children. Furthermore, there was no false positive results, since all 106 children with long-term follow up with negativization of antibodies have had negative PCR at birth and at 1 month of age. If our results are confirmed in larger prospective studies, blood PCR might be a very useful tool to rule out CD early on in children born to *T*. *cruzi* infected mothers. It would be of particular interest in our setting where a high proportion of children are lost to follow-up during the first months of life.

Antibodies negativization confirmed by serologic testing is recommended starting at age of 9 months, although antibodies may not actually become undetectable until age of 12 months. In our case, the average age at which the antibodies were negative in non-infected patients was 10.5 months. According to our results, we suggest that serology should not be performed before 10 months of age, to avoid unnecessary blood samples.

New advances in the treatment of this disease are also necessary. There are two approved drugs: benznidazole (of choice) and nifurtimox (alternative treatment); both are more effective in the acute phase of the disease; and more effective and better tolerated at a younger age. The young infant’s treatment presents a cure rate close to 100% [4].

These drugs may cause side effects, most frequently gastrointestinal, cutaneous and hematologic, so the treatment requires monitoring of patients. These reactions are more frequent over the age of 7 years [16].

Our study presents some limitations, first of all because it is a retrospective descriptive study. Due to the sample size, the confidence interval of the VT rate is wide. Performance of multicenter studies with greater number of patients or studies with a longer period of follow-up would be considered to obtain more consistent results. Another limitation is that we did not perform the two parasitological determinations (microhaematocrit and PCR) in all newborns (only in the 30,4% of them) so a comparison between both techniques can not be done.

Nevertheless, our series provides important data regarding the estimation of the VT rate in a non-endemic area and that reinforces, in conclusion, the need to carry out a universal screening in the population coming from endemic areas. An early diagnosis and an effective and better tolerated treatment reduce the prevalence of the disease and its long-term complications.

Due to the non-negligible percentage of losses in follow-up of these patients, it seems relevant that we have not obtained any false negative or false positive from the PCR. If these findings are confirmed, it could imply avoiding unnecessary follow-ups with serological studies in the future. However, the design of the study and the results obtained do not have the strength to make possible to modify the current screening method, it would be necessary to perform prospective studies with a larger sample size and designed specifically for this purpose.

## Supporting information

S1 File Database. Studied variables data.
(XLSX)
